# Integrin-linked kinase control dental pulp stem cell senescence via the mTOR signaling pathway

**DOI:** 10.1093/stmcls/sxae047

**Published:** 2024-09-02

**Authors:** Lu Chen, Xiping Wang, Sha Tian, Linxi Zhou, Li Wang, Xiaohan Liu, Zihan Yang, Guiqiang Fu, Xingguang Liu, Chen Ding, Duohong Zou

**Affiliations:** Department of Oral Surgery, Shanghai Ninth People’s Hospital, College of Stomatology, Shanghai Jiao Tong University School of Medicine; National Clinical Research Center for Oral Diseases Shanghai Key Laboratory of Stomatology and Shanghai Research Institute of Stomatology, Shanghai 200011, People’s Republic of China; Institute of Stomatology, School and Hospital of Stomatology, Wenzhou Medical University, Wenzhou 325027, People’s Republic of China; State Key Laboratory of Genetic Engineering, School of Life Sciences, Human Phenome Institute, Fudan University, Shanghai 200433, People’s Republic of China; Department of Orthodontics, Shanghai Ninth People’s Hospital, Shanghai Jiao Tong University School of Medicine; College of Stomatology, Shanghai Jiao Tong University; National Center for Stomatology; National Clinical Research Center for Oral Diseases; Shanghai Key Laboratory of Stomatology, Shanghai, People’s Republic of China; Institute of Stomatology, School and Hospital of Stomatology, Wenzhou Medical University, Wenzhou 325027, People’s Republic of China; Department of Oral Surgery, Shanghai Ninth People’s Hospital, College of Stomatology, Shanghai Jiao Tong University School of Medicine; National Clinical Research Center for Oral Diseases Shanghai Key Laboratory of Stomatology and Shanghai Research Institute of Stomatology, Shanghai 200011, People’s Republic of China; Institute of Stomatology, School and Hospital of Stomatology, Wenzhou Medical University, Wenzhou 325027, People’s Republic of China; Stomatology Hospital and College, Key Laboratory of Oral Diseases Research of Anhui Province, Anhui Medical University, Hefei, People’s Republic of China; National Key Laboratory of Medical Immunology & Institute of Immunology, Second Military Medical University, Shanghai 200433, People’s Republic of China; State Key Laboratory of Genetic Engineering, School of Life Sciences, Human Phenome Institute, Fudan University, Shanghai 200433, People’s Republic of China; Department of Oral Surgery, Shanghai Ninth People’s Hospital, College of Stomatology, Shanghai Jiao Tong University School of Medicine; National Clinical Research Center for Oral Diseases Shanghai Key Laboratory of Stomatology and Shanghai Research Institute of Stomatology, Shanghai 200011, People’s Republic of China; Institute of Stomatology, School and Hospital of Stomatology, Wenzhou Medical University, Wenzhou 325027, People’s Republic of China

**Keywords:** HDPSCs, cellular senescence, osteogenesis, ILK, proteomic

## Abstract

Human dental pulp stem cells (HDPSCs) showed an age-dependent decline in proliferation and differentiation capacity. Decline in proliferation and differentiation capacity affects the dental stromal tissue homeostasis and impairs the regenerative capability of HDPSCs. However, which age-correlated proteins regulate the senescence of HDPSCs remain unknown. Our study investigated the proteomic characteristics of HDPSCs isolated from subjects of different ages and explored the molecular mechanism of age-related changes in HDPSCs. Our study showed that the proliferation and osteogenic differentiation of HDPSCs were decreased, while the expression of aging-related genes (p21, p53) and proportion of senescence-associated β-galactosidase (SA-β-gal)-positive cells were increased with aging. The bioinformatic analysis identified that significant proteins positively correlated with age were enriched in response to the mammalian target of rapamycin (mTOR) signaling pathway (ILK, MAPK3, mTOR, STAT1, and STAT3). We demonstrated that OSU-T315, an inhibitor of integrin-linked kinase (ILK), rejuvenated aged HDPSCs, similar to rapamycin (an inhibitor of mTOR). Treatment with OSU-T315 decreased the expression of aging-related genes (p21, p53) and proportion of SA-β-gal-positive cells in HDPSCs isolated from old (O-HDPSCs). Additionally, OSU-T315 promoted the osteoblastic differentiation capacity of O-HDPSCs in vitro and bone regeneration of O-HDPSCs in rat calvarial bone defects model. Our study indicated that the proliferation and osteoblastic differentiation of HDPSCs were impaired with aging. Notably, the ILK/AKT/mTOR/STAT1 signaling pathway may be a major factor in the regulation of HDPSC senescence, which help to provide interventions for HDPSC senescence.

## Graphical Abstract

**Graphical Abstract:**
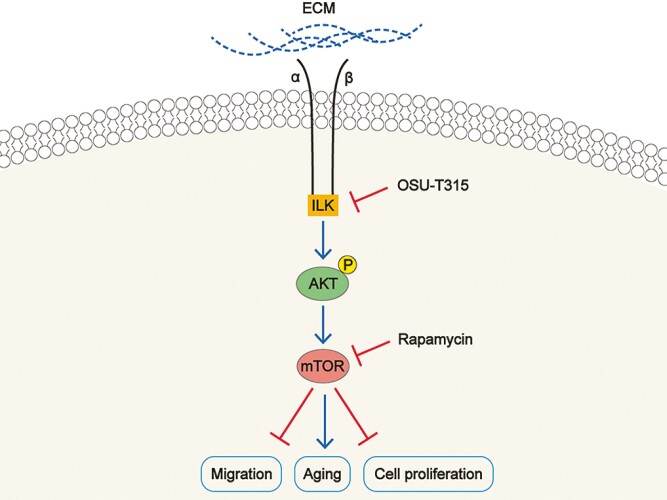

**Significance statement**

This study established a comprehensive landscape of human dental pulp stem cells (HDPSCs) at the proteomic level and revealed that the senescence of HDPSCs is a multifaceted process that involves DNA replication, differentiation, migration, immunity, and autophagy. Elucidating the molecular mechanisms of HDPSC senescence is vital for improving the therapeutic effects of HDPSC-mediated regenerative medicine. Our results suggested that the ILK/AKT/mTOR/STAT1 signaling pathway may be the main factor regulating the senescence of HDPSC. This study provides a strategy to alleviate aging-associated phenotype of HDPSCs and promote osteogenesis of O-HDPSCs.

## Introduction

Dental pulp stem cells (DPSCs) have the potential of multilineage differentiation, such as osteogenesis, neurogenesis, dentinogenesis, and chondrogenesis, which makes them as promising sources for regenerative medicine.^1,2^ Recently, multiple studies have demonstrated that DPSCs are safe and efficacious for complete regenerative endodontics.^3,4^ Moreover, Lee et al reported that combined with xenografts, DPSCs showed excellent bone regeneration, like the regenerative capacity of bone marrow mesenchymal stem cells (BMSCs) in vivo.^5^

However, similar to BMSCs, DPSCs show an age-dependent decline in proliferation and multilineage differentiation.^6-8^ Senescence, a critical factor affecting DPSCs, plays an important role in maintaining the proliferation and plasticity of stem cell.^9^ Activation of chronic DNA damage responses, shortening of telomeres, and upregulation of lysosomal proteins is typical phenotypes of cellular senescence.^10,11^ It was previously demonstrated that downregulation of ROR2 accelerated the senescence of DPSCs via activation of the MSX2/NSUN2/p21 axis.^12^ Moreover, the changes in cell quiescence and differentiation potential are mainly due to mitochondrial damage caused by reduced autophagic activity.^13^ Additionally, the expression of p16^INK4A^ and p21 was significantly increased with aging, affecting the proliferative and tissue regenerative capacity of DPSCs.^14^ Further studies are needed to elucidate the molecular mechanism of age-related changes in DPSCs.

Previous studies have reported that the activation of the mammalian target of rapamycin (mTOR) signaling pathway causes excessive protein synthesis, resulting in impaired protein balance and accelerated aging.^15^ mTORC1 is a negative regulator of autophagy.^16^ Rapamycin, an inhibitor of mTOR, is a major life-extending compound.^10,17^ Although the critical role of mTOR signaling in mammalian aging is known, its specific mechanisms in HDPSC senescence needs to be further elucidated. Integrin-linked kinase (ILK) is mostly localized to the cell membrane and plays an important role in cell proliferation, migration, and differentiation.^18-20^ Protein kinase B (Akt), a downstream target of ILK, is involved in regulating the mTOR signaling pathway.^21-23^ Whether the ILK and mTOR signaling pathways are involved in regulating the senescence of HDPSC remains unknown.

Our study identified that mTOR signaling pathway was one of the significantly enriched genes positively correlated with age by proteomic analysis. Inhibition of both ILK and mTOR signaling pathways alleviated HDPSC senescence. Furthermore, OSU-T315, an inhibitor of ILK, promoted osteoblastic differentiation of HDPSCs isolated from old subjects (O-HDPSCs) in vitro and in vivo. Our results provided interventions for HDPSC senescence and improved the tissue regenerative efficacy of O-HDPSCs.

## Materials and Methods

### HDPSC isolation and culture

Dental pulp was obtained from surgically extracted intact human third molars ranging in age from 16 to 70 years (*n* = 25). Details of different age donors were recorded (Supplementary Appendix Table 1). This study was approved by the Biomedical Ethics Committee of Ninth People’s Hospital, Shanghai Jiao Tong University School of Medicine (Project agreement SH9H-2019-T167-2) and informed consent forms were obtained from the participants or their guardians.

Before separating pulp tissue, the teeth were rinsed with phosphate-buffered saline for 3 times. The collected pulp tissue was minced and digested with 4 mg/mL dispase (Gibco, Grand Island, NY, USA) and 3 mg/mL collagenase type I (Invitrogen Life Technology, Carlsbad, CA, USA) at 37 °C for 1 hour. Then, the digested single-cells were collected and placed in a 6-cm culture dish with α-modified minimum essential medium (α-MEM) containing 1% penicillin-streptomycin (Beyotime, China) and 20% fetal bovine serum (FBS; Bio-Ind, Israel). In this study, passages 3-5 of HDPSCs were used.

### Cell proliferation and migration assay

HDPSCs were seeded in 96-well plates (5 × 10^3^ cells/well). Cell Counting Kit-8 (CCK8; Dojindo, Japan) was used to detect the rate of cell proliferation. 1 × 10^5^ cells/mL cell suspension was prepared with serum-free medium. In the transwell (BD Matrigel, 3422, Costar) migration assay, 500 μL culture medium containing FBS was added into the lower chamber, and 200 μL cell suspension was added into the upper chamber. After incubation for 24 hours, crystal violet ammonium oxalate solution (Solarbio, China) was added for staining, and then photographed under microscope.

### Senescence‐associated‐β galactosidase assay

Senescent cells were detected using a β-galactosidase staining kit (Solarbio, China), according to the instructions for using the reagent. The count was observed under an ordinary light microscope. For some experiments, O-HDPSCs were determined for SA-β-Gal staining in the presence of Rapamycin (AY-22989, Selleck) or OSU-T315 (ILK-IN-2, Selleck). When reached 60% confluence, O-HDPSCs were treated with serial concentrations of Rapamycin (0, 100, and 200 nM) or OSU-T315 (0, 250, and 500 nM) for 3 days.

### Osteogenic and neurogenic induction

HDPSCs were seeded in 6-well plates (1 × 10^5^ cells/well). When HDPSCs reached 60%-70% confluence, the conventional medium was replaced with osteogenic differentiation medium (Cyagen, USA). After 21 days of culture, the cells were fixed with 4% paraformaldehyde and stained with 1% Alizarin Red S solution for 10 minutes (Cyagen).

HDPSCs were seeded in 6-well plates (1 × 10^5^ cells/well). When HDPSCs reached 50% confluence, the conventional medium was replaced with neurogenic differentiation medium (Gibco, Life Technologies, Carlsbad, CA) consisting of 1% B27 (Gibco, Life Technologies), 1% penicillin-streptomycin, 20 ng/mL epidermal growth factor (Thermo), and 40 ng/mL fibroblast growth factor 2 (Thermo).

### Cell migration assay

After pretreatment with inhibitors for 3 days, HDPSCs were seeded in 6-well plates. When HDPSCs reached 90% confluence, a 200-µL pipette tip was used to scratch the monolayers. Images were acquired every 12 hours.

### Western blotting

HDPSCs were lysed with Cell lysis buffer (#9803, CST). Bradford assay (Sangon Biotech, China) was used to assess protein concentration. Twenty micrograms of proteins were separated by SDS-polyacrylamide gel and then transferred to polyvinylidene difluoride (PVDF; Millipore, USA) membrane. After blocking with 5% nonfat milk, the membranes were incubated with primary antibodies p21(1:1000, 2947S, CST), STAT1(1:1000,14994T, CST), mTOR (1:1000, 2983S,CST), p-mTOR (1:1000, 5536S, CST), p53 (1:1000, ab26, Abcam), STAT6 (1:1000, ab32520, Abcam), BAZ1B (1:1000, ab51256, Abcam), SMARCA1 (1:1000, ab175226, Abcam), ILK (1:1000, ab52480, Abcam), GAPDH (1:5000, ab8245, Abcam), Akt (1:1000, MAB2055, R&D Systems), and p-Akt (1:1000, AF887, R&D Systems) overnight at 4 °C. The membranes were incubated with suitable secondary antibodies (1:5000) and the proteins were visualized by a chemiluminescence (ECL; Millipore, USA, WBKLS0500) and imaging system (Bio-Rad, USA).

### Real-time polymerase chain reaction

TRIzol (TaKaRa) was used to extract total RNA from the HDPSCs. cDNA was synthesized using the SuperScript Reverse Transcriptase Kit (TaKaRa) to analyze mRNA expression. The relative levels of the genes were normalized with GAPDH and analyzed using the equation 2^−∆∆CT^.^24^ qPCR was performed with specific primers (Supplementary Appendix Table 2).

### Protein extraction and trypsin digestion

HDPSCs was mixed with urea (8 M) for reduction of disulfide bridges, cysteine alkylation, and protein denaturation at 95 °C for 10 minutes. The crude exact was then clarified by centrifugation at 16,000 × *g* for 10 min and the supernatants were loaded into 10 kDa Microcon filtration devices (Millipore) and centrifuged at 12,000 × *g* for 20 min and washed twice with urea lysis buffer (8 M urea, 100 mM Tris-HCl, pH 8.0), and twice with 50 mM NH_4_HCO_3_. Then the samples were digested using trypsin at an enzyme to protein mass ratio of 1:25 overnight at 37 °C. Peptides were extracted and dried (SpeedVac, Eppendorf) (PMID: 31495056).

### Label-free-based MS quantification of proteins

The one-stop proteomic cloud platform “Firmiana” was further used for protein quantification. Identification results and the raw data from mzXML file were loaded. Then for each identified peptide, the XIC (extracted-ion chromatogram) was extracted by searching against the MS1 based on its identification information, and the abundance was estimated by calculating the area under the extracted XIC curve (AUC). For protein abundance calculation, the nonredundant peptide list was used to assemble proteins following the parsimony principle. Then, the protein abundance was estimated with a traditional label-free, intensity-based absolute quantification (iBAQ) algorithm, which divided the protein abundance (derived from identified peptides’ intensities) by the number of theoretically observable peptides. Then the fraction of total (FOT), a relative quantification value which was defined as a protein’s iBAQ divided by the total iBAQ of all identified proteins in one experiment, was calculated as the normalized abundance of a particular protein among experiments. Finally, the FOT was further multiplied by 10^6^ for the ease of presentation and FOTs less than 10^6^ were replaced with 10^6^ to adjust extremely small values.

### Proteomic analysis and bioinformatics analysis

Proteomic analysis of samples from Young, Middle-aged, and Old donors was performed at the Institute of Human Phenome, Fudan University, Shanghai, China. Raw MS files were processed with the “Firmiana” (a one-stop proteomic cloud platform) against the RefSeq protein database of the National Center for Biotechnology Information. The ConsensusClusterPlus R package (Wilkerson and Hayes, 2010) was used for consensus clustering of proteins expressed in all samples (*n* = 1822). ConsensusPathDB was utilized for pathway enrichment analysis. Weighted gene co-expression network analysis (WGCNA) was applied to the proteins that were used for proteomic subtyping.

### Rat calvarial bone defects reconstructed by HDPSCs scaffolds

Animal experiments were approved by the Ethics Committee of Shanghai Ninth People’s Hospital (Project agreement SH9H-2019-A720-1). Efforts were made to minimized animal discomfort or pain. Eighteen 5-8 weeks old male Sprague-Dawley rats were used to evaluate bone regeneration in vivo. Each rat was treated independently as one experimental unit. After the calvarial bone was exposed, a critical-size defect was created with an 8-mm dental trephine. The commercialized collagen sponge (6 mm × 6 mm × 3 mm, Helistat 1690ZZ) was used as scaffold to seed with HDPSCs. The scaffold seeded with Y-HDPSCs (Y-HDPSCs group), the scaffold without HDPSCs (blank group), and the scaffold seeded with 5 × 10^5^ O-HDPSCs pretreated with 0 nM OSU-T315 (0 nM OSU-T315 group) or 500 nm OSU-T315 (500 nM OSU-T315 group) were inserted into each defect. Six in every group. After 8 weeks, animals were euthanized with CO_2_ and the repaired skulls were removed. Micro-CT (Bruker Skyscan1176) was used for calvarial bone defect repair examination. Bone mineral density (g/cc) was measured and analyzed. Sections (5 μm) were prepared with a microtome for hematoxylin-eosin (H&E) staining.

### Statistical analysis

All results represented 2 or more independent experiments. All data are shown as the mean ± SD. Two-tailed Student’s *t* test was used to indicate the differences between 2 groups. One-way ANOVA analysis was used to indicate the differences among more than 2 groups. A *P-*value < .05 was considered statistically significant.

## Results

### Features of HDPSCs with aging

**Figure 1.**
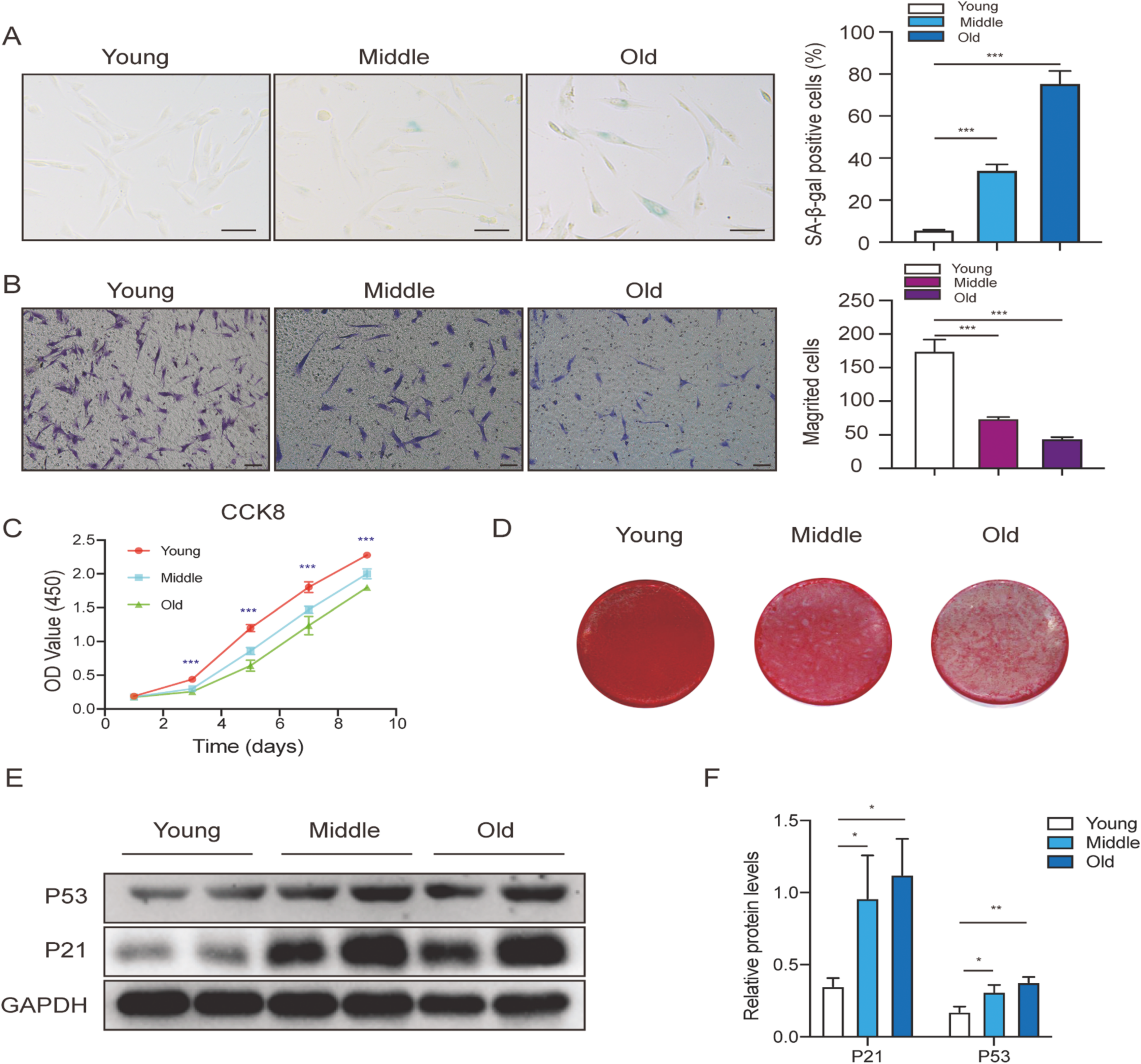
Characteristics of HDPSCs isolated from donors of different ages. (A) Representative images of SA-β-gal staining of HDPSCs in the 3 age groups. Quantitative analysis of the ratio of SA-β-gal-positive cells in the 3 groups (*n* = 3). Scale bar = 100 μm. (B) Representative images of migrated HDPSCs in the 3 age groups. Quantitative analysis of migrated cells (*n* = 3). Scale bar = 50 μm. (C) Results of the CCK-8 assay in different age groups (*n* = 3). (D) Representative images of ARS of HDPSCs in the 3 age groups induced in an osteogenic induction medium for up to 21 days. (E and F) Western blot for P21, P53, and GAPDH (loading control) was performed from HDPSCs in the 3 age groups. *P < .05, **P < .01, ***P < .001.

SA-β-gal staining assays revealed that the SA-β-gal activity of O-HDPSCs was remarkably higher than those of HDPSCs isolated from young subjects (Y-HDPSCs) (Figure 1A). Y-HDPSCs exhibited a stronger migrative capacity than O-HDPSCs (Figure 1B). CCK-8 assays revealed that the proliferation of HDPSCs was gradually declined with aging (Figure 1C). Furthermore, O-HDPSCs exhibited impaired osteogenic differentiation potential, as shown by Alizarin red staining (ARS) (Figure 1D). Additionally, western blotting showed that the expression of senescence-related genes (p21 and p53) was elevated with aging (Figure 1E and F). These results revealed that the capacity of proliferation, migration, and osteogenic differentiation was impaired, whereas the expression of senescence-related genes (p21 and p53) and SA-β-gal activity was increased with aging. Representative images of 3 groups (Figure 1A, B and D) were taken from individuals aged 16, 36, and 69. All results represented 3 biological replicates.

### HDPSCs samples and clustering of HDPSCs on protein abundance

The schematic diagram of proteomics experimental design is shown in Supplementary Appendix Figure 1A. The proteomics measurement and proteomics data analysis were performed on all samples, and a total of 6348 protein groups were obtained at the protein and peptide levels with a 1% false discovery rate Supplementary Appendix Figure 1B). On average, the HDPSC proteome of each sample had 3631 protein groups, with a minimum of 3012 and a maximum of 3968 (Supplementary Appendix Figure 1C and D). The samples showed a relatively consistent mean distribution for the protein abundances (Supplementary Appendix Figure 1E), confirming high technical reproducibility.

**Figure 2.**
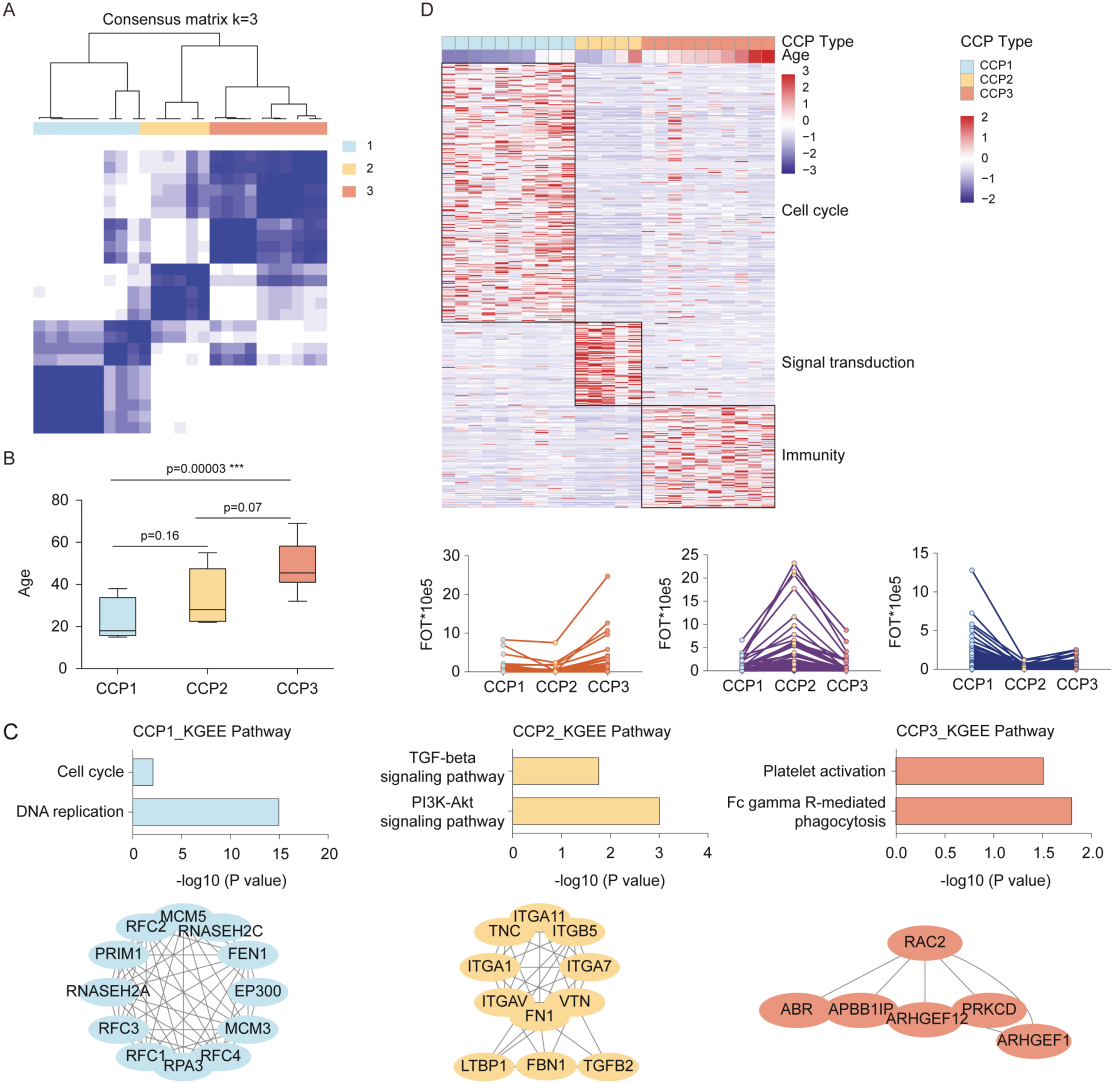
Molecular Subtyping of HDPSCs. (A) Consensus matrices of identified clusters (*k* = 3). (B) Boxplot of age in CCP subtypes. (C) Differentially expressed proteins in CCP subtypes and their associated biological pathways (left, > 1.5-fold, *t* test, *P* < .05). Line plots of selected gene sets with up- or downregulation trends in CCP subtypes (right, > 1.5-fold, *t* test, *P* < .05). (D) Analysis of significantly differentially regulated pathways (up) and protein interaction network (down) in CCP subtypes.

Consensus clustering^25^ identified 3 proteomic subtypes significantly associated with age (Figure 2A and B). Three of the proteomic clusters (CCP1-3) were evident: CCP1 corresponding to Y-HDPSCs contained the most differentially expressed proteins (264, >1.5-fold, *P* < .05), which were enriched in cell cycle-related processes; CCP2 corresponding to HDPSCs isolated from middle-aged subjects (M-HDPSCs) contained the fewest differentially expressed proteins (89, >1.5-fold, *P* < .05), which were involved in the signal transduction-related processes; and CCP3 corresponding to O-HDPSCs was characterized by the enrichment of proteins involved in the immunological process (104, > 1.5-fold, *P* < .05; Figure 2C). Thus, HDPSCs could be subtyped as cell cycle (CCP1, *n* = 10), signal transduction (CCP2, *n* = 5), and immunity enrichment subtype (CCP3, *n* = 10) based on their altered proteomic patterns alone (Figure 2C and D). Top 10 KEGG pathways for each CCP were shown in Supplementary Appendix Table 3.

### Association of proteomic subtypes of HDPSCs with cell phenotypic characteristics

**Figure 3.**
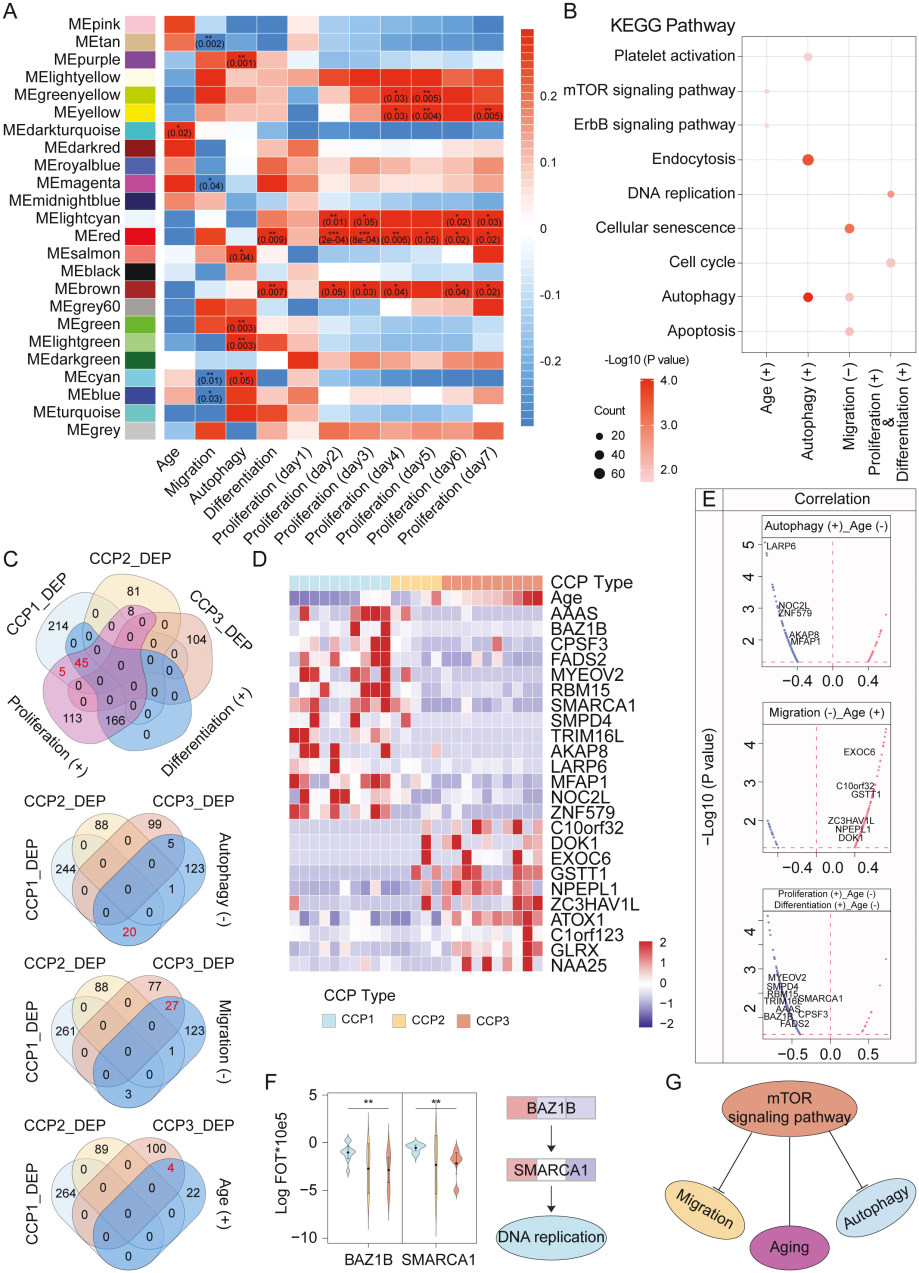
WGCNA analysis. (A) Correlation of WGCNA-derived protein modules with phenotypes for HGSC. The correlations are annotated by cell color with the p value corresponding to the significance of the association. (B) Enrichment of KEGG ontologies in the WGCNA-derived modules. The enrichment is denoted by a dot colored according to the significance (*q* value) of association and with a size scaled by proteins mapped to a particular ontology over the total number of proteins in the module (GeneRatio). (C) Venn diagram summary of the number of proteins in CCP_DEP and WGCNA-derived modules (differentiation, proliferation, migration, autophagy, and age). (D and E) A list of signature proteins that were differentially expressed in CCP1-3 (> 1.5-fold, *t* test, *P* < .05). Nine proteins were significantly associated with proliferation and differentiation. Six proteins were significantly associated with migration. Six proteins were significantly associated with autophagy. (F) Proteins (BAZ1B and SMARCA1, left) in pathways (DNA replication, right) that were differentially expressed in the 3 proteomic subtypes. (G) Schematic diagram of signaling pathway regulation.

To investigate the correlation between proteomic subtypes of HDPSCs and cellular phenotype, we performed WGCNA and identified 14 co-expression modules (Figure 3A).^26^ These modules were significantly associated age (*P* < .05). The enrichment of Kyoto Encyclopedia of Genes and Genomes (KEGG) in the WGCNA-derived modules is shown in Figure 3B. Significant proteins positively correlated with differentiation and proliferation were enriched in response to the DNA replication and cell cycle; significant proteins positively correlated with age were enriched in response to the mTOR signaling pathway and ErbB signaling pathway; significant proteins positively correlated with autophagy were enriched in response to endocytosis, autophagy, and platelet activation; and significant proteins negatively correlated with migration were enriched in the response to cellular senescence, autophagy, and apoptosis. Moreover, we observed that 50 specific highly expressed proteins in CCP1 overlapped with the proteins significantly positively correlated with differentiation and proliferation; 20 specific highly expressed proteins in CCP3 overlapped with the proteins significantly negatively correlated with autophagic ability; twenty-seven proteins specifically highly expressed in CCP3 overlapped with the proteins significantly negatively correlated with migration; and 4 specific highly expressed proteins in CCP3 overlapped with the proteins significantly positively correlated with age (Figure 3C). In addition, 9 proteins were positively correlated with differentiation and proliferation, but negatively correlated with age; 5 proteins significantly positively correlated with autophagy were negatively correlated with age; and 6 proteins significantly negatively correlated with migration ability showed a positive correlation with age (Figure 3D and E). The representative differentially expressed proteins associated with proliferation are shown in Figure 3F. BAZ1B and SMARCA1 were well correlated with DNA replication. Notably, the mTOR signaling pathway may be a major factor in the negative correlation between aging and autophagy (Figure 3G).

### Proteomic analysis of pathways associated with autophagy

**Figure 4.**
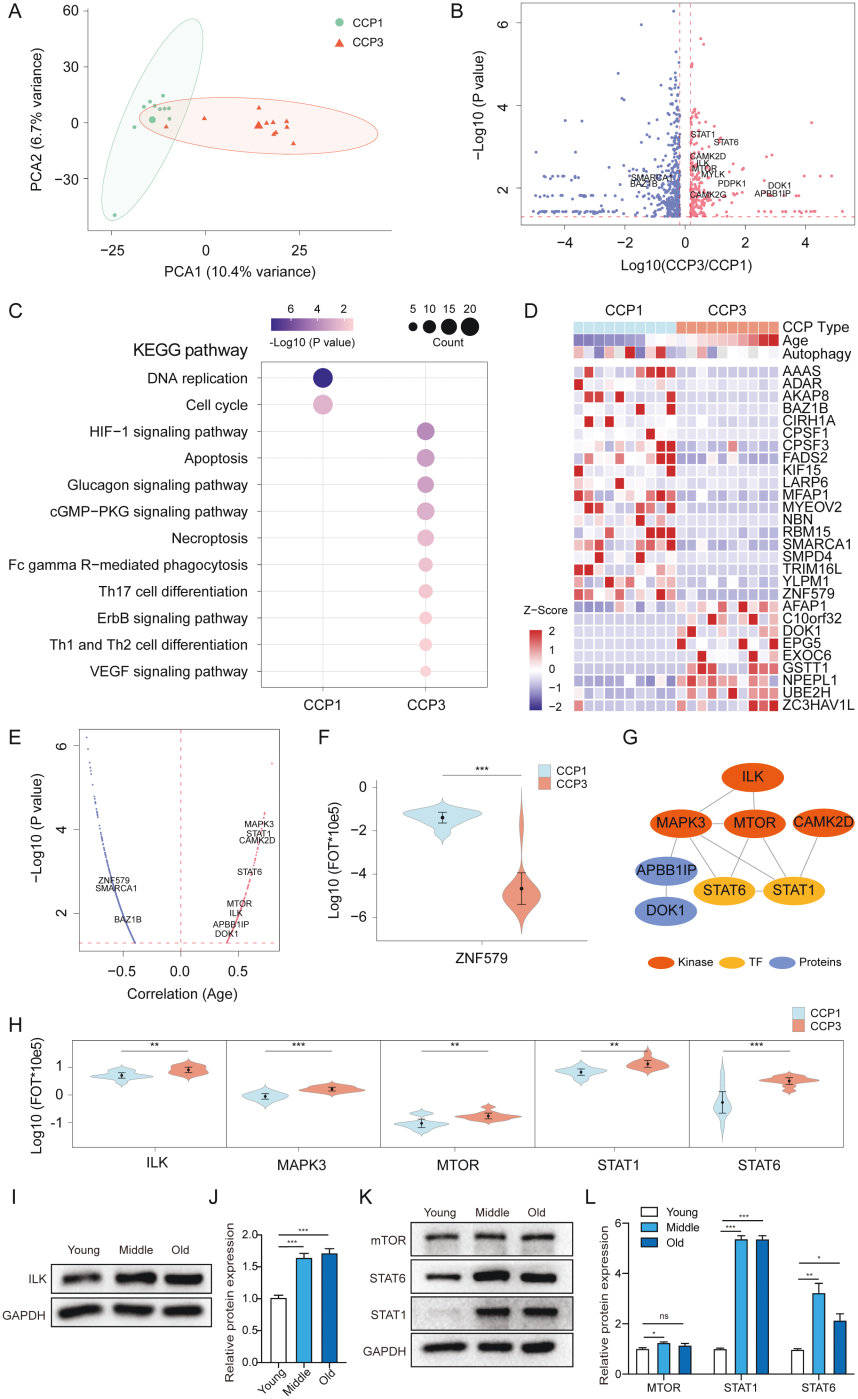
Proteomics features of CCP1 and CCP3 in HDPSCs. (A) Principal component analysis of 1822 proteins in 20 samples. (B) Volcano plot indicating proteins overexpressed in CCP1 or CCP3. Red and blue colors indicate upregulated and downregulated proteins in CCP3, respectively (>1.5-fold, *t* test, *P* < .05). (C) Pathways enriched for the 471 proteins upregulated in CCP1 and 236 proteins upregulated in CCP3. (D and E) Heatmap of signature proteins that were differentially expressed in CCP1 and CCP3 (> 1.5-fold, *t* test, *P* < .05). Nineteen proteins were significantly negatively associated with age. Nine proteins were significantly positively associated with age. (F-H) Protein abundances of ZNF579, ILK, MAPK3, MTOR, STAT1, and STAT6 in CCP1 and CCP3 (>1.5-fold, *t* test, *P* < .05) and their network. (I) Expression of ILK in Y-DPSCs, M-HDPSCs, and O-HDPSCs. (J) Quantificative analysis of protein levels. Data are presented as the mean ± SD, *n* = 3. (K) Expression of the mTOR signaling pathway-related genes mTOR, STAT1, and STAT3 in Y-DPSCs, M-HDPSCs, and O-HDPSCs. (L) Quantificative analysis of protein levels. Data are presented as the mean ± SD, *n* = 3. *P < .05, **P < .01, ***P < .001.

Among the 6348 proteins identified by liquid chromatography-tandem MS (LC-MS/MS), 6103 were quantified in CCP1 and CCP3. Principal component analysis revealed a clear boundary between the CCP1 and CCP3 proteomes (Figure 4A), indicating a differential proteomic landscape by age (Figure 2b, >1.5-fold, *P* < .05). There were 707 proteins differentially expressed between CCP1 and CCP3 (*P* < .05). Among them, 236 were upregulated and 471 were downregulated in CCP3 (Figure 4B). KEGG pathway enrichment indicated that proteins in CCP3 were enriched in immunity pathways such as the HIF − 1 signaling pathway, ErbB signaling pathway, and Th17 cell differentiation, whereas those in CCP1 were enriched in cell proliferation pathways such as DNA replication and cell cycle (Figure 4C). Nine proteins significantly positively correlated with age had specific high expression in CCP3 (Figure 4D). Notably, 19 proteins significantly negatively correlated with age had specific high expression in CCP1 (Figure 4D). In addition, 4 kinases (CAMK2D, ILK, MAPK3, and MTOR) and 2 transcription factors (STAT1 and STAT6) with higher expression in CCP3 were significantly positively correlated with age, whereas that with higher expression in CCP1 (BAZ1B, SMARCA1, and ZNF579) were significantly negatively correlated with age (Figure 4E and F). To study the relationship between autophagy and age in HDPSCs, we constructed a kinase-TF network map using CCP3 highly expressed protein. As shown in Figure 4G and H, STAT1 and STAT6 may be regulated by the phosphorylation signal of mTOR kinase to inhibit autophagy and cause aging. Next, we validated by western blotting that several of enriched proteins (ILK, mTOR, STAT1, and STAT3) upregulated with aging (Figure 4I-L). Overall, our proteomics data provided additional insights into the phenotypic characteristics of HDPSCs.

### ILK cooperates with mTOR to regulate HDPSC senescence

**Figure 5.**
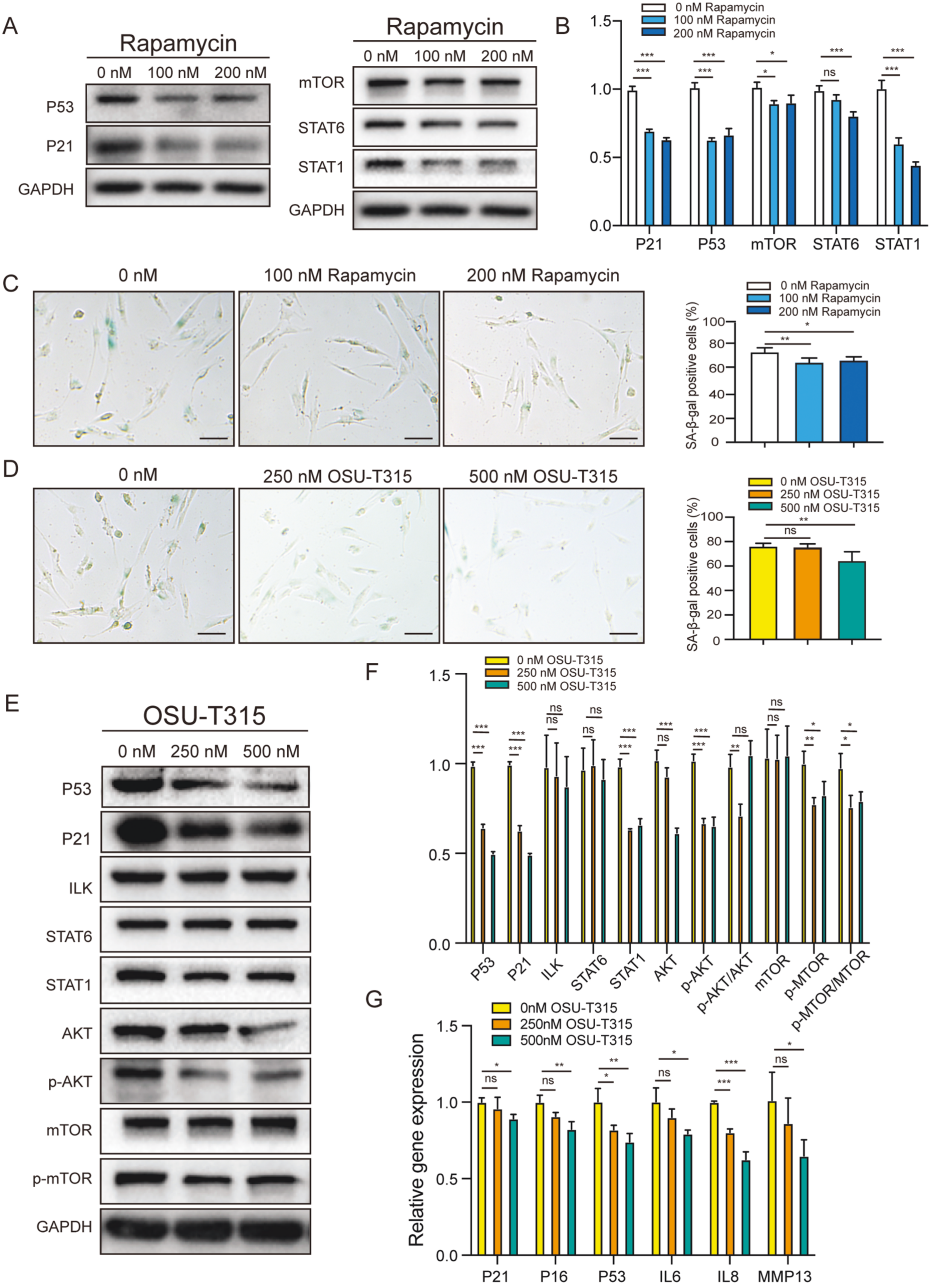
Amelioration of senescence of O-HDPSCs by inhibition of integrin-linked kinase (OSU-T315) and mTOR (rapamycin). (A and B) O-HDPSCs treated with serial concentrations of rapamycin for 3 days are subjected to western blot analysis. (C) Representative images of SA-β-gal staining of HDPSCs treated with serial concentration of rapamycin. Quantitative analysis of the number of SA-β-gal-positive cells (*n* = 3). Scale bar = 50 μm. (D) Representative images of SA-β-gal staining of HDPSCs treated with serial concentration of OSU-T315. Quantitative analysis of the number of SA-β-gal-positive cells (*n* = 3). Scale bar = 50 μm. (E and F) O-HDPSCs treated with serial concentrations of OSU-T315 for 3 days were subjected to western blot analysis. (G) qPCR analysis of stress response genes in the p53 pathway, senescence-associated interleukin-6, interleukin-8, and metalloprotease MMP13 after treatment with OSU-T315 (*n* = 3). *P < .05, **P < .01, ***P < .001.

Our studies showed that 2 kinases (ILK and mTOR) and 2 transcription factors (STAT1 and STAT6) were more highly expressed in CCP3 (O-HDPSCs; Figure 4E). Therefore, we next investigated whether inhibition of the ILK and mTOR was involved in regulating HDPSC senescence. Inhibition of mTOR signaling pathway with rapamycin induced decreased expression of senescence-related genes (p21 and p53) and SA-β-gal activity (Figure 5A, B, and C). Treatment with OSU-T315 (an inhibitor of ILK) induced decreased expression of senescence-related genes (p21 and p53) and SA-β-gal activity (Figure 5D, E, and F). These results suggested that rapamycin and OSU-T315 could ameliorate senescence of O-HDPSCs. The expression level of STAT1 decreased remarkably after treatment with rapamycin or OSU-T315 (Figure 5A and E). Inhibition of both the ILK and mTOR signaling pathways affected STAT1 signaling activation, which occurred during HDPSC senescence (Figures 5A, E and 4K). After inhibition of ILK, the expression levels of p-AKT and p-mTOR decreased significantly, while the expression levels of AKT and mTOR did not change much (Figure 5E and F). Consistent with reported research, inhibition of ILK repressed phosphorylated activation of AKT and mTOR. Furthermore, qPCR analysis revealed that the mRNA levels of senescence-related genes (p16^INK4A^, p21, and P53), senescence-associated interleukin-6, interleukin-8, and metalloprotease MMP13 were decreased after pretreatment with OSU-T315 (Figure 5G). Our results indicated that the ILK/AKT/mTOR/STAT1 signaling pathway may be the main factor regulating the senescence of HDPSC.

### Cell function changes of O-HDPSCs after pretreatment with inhibitors

After pretreatment with inhibitors (OSU-T315 or Rapamycin) for 3 days, O-HDPSCs were seeded for cell proliferation and migration assay. The results showed that pretreatment with inhibitors (OSU-T315 or Rapamycin) did not significantly affect the proliferation and migration capacity of HDPSCs (Supplementary Appendix Figure 2 A, B, E, and F). O-HDPSCs pretreated with OSU-T315 for 3 days showed elevated mRNA expression of neurogenic markers (SOX2, GFAP, and GAP43) after induced by neurogenic differentiation medium for 9 days (Supplementary Appendix Figure 2 C). We tried to evaluate a putative synergistic effect between inhibitors. The 2 inhibitors may have a putative synergistic on alleviating HDPSC senescence (Supplementary Appendix Figure 2F). The mRNA expression of senescence-associated interleukin-6 and metalloprotease MMP13 was increased with aging (Supplementary Appendix Figure 2G).

### OSU-T315 promotes osteogenesis of O-HDPSCs in vitro and vivo

**Figure 6.**
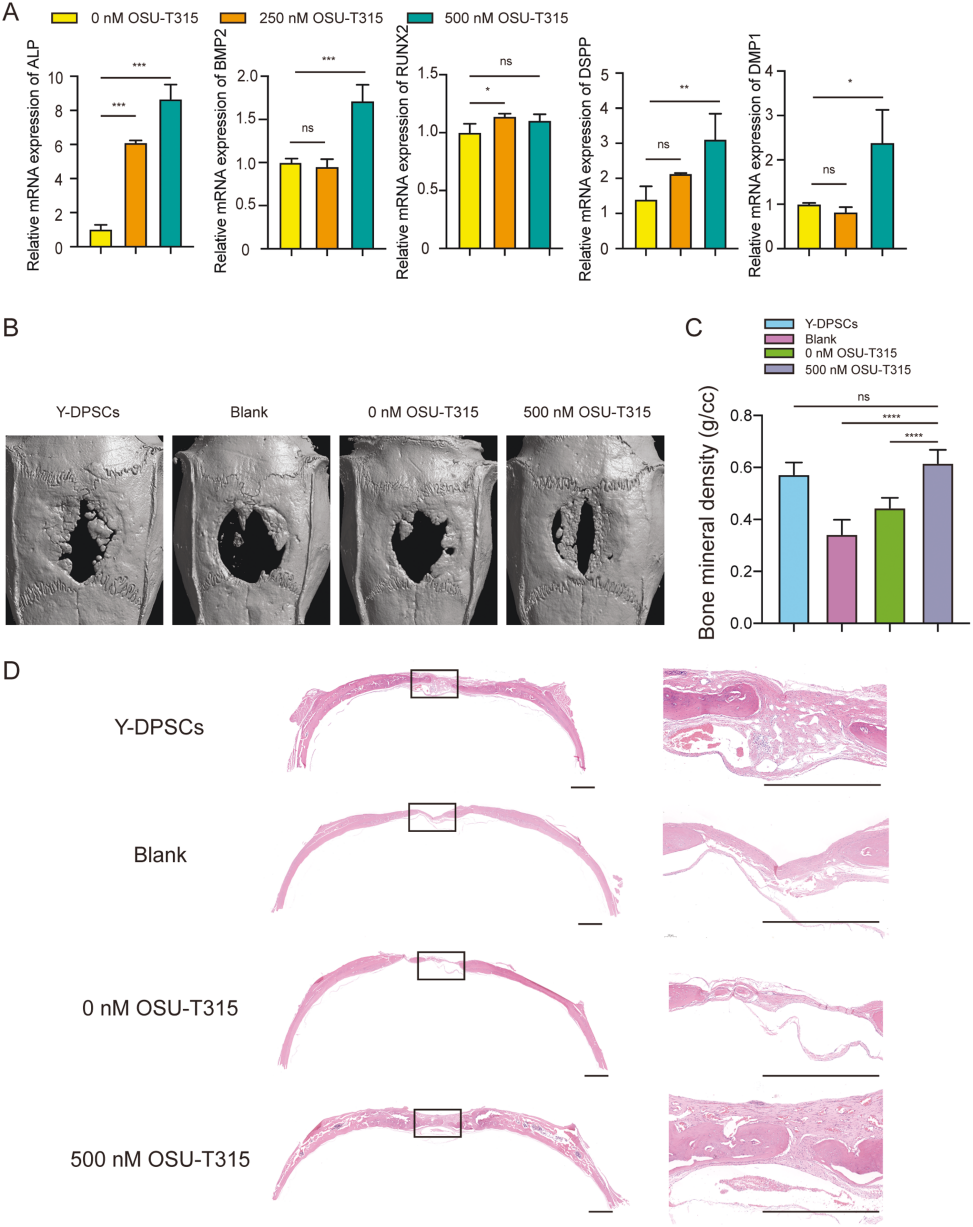
OSU-T315 promotes osteogenesis of O-HDPSCs in vitro and vivo. (A) Quantitative RT-PCR results of mRNA expression of ALP, BMP2, RUNX2, DMP1, and DSPP in O-HDPSCs pretreated with and without OSU-T315 induced in an osteogenic induction medium for up to 9 days (*n* = 3). (B) Representative images of micro-CT reconstruction of rat calvarial bone defect at 8 weeks. (C) Quantitative micro-CT analysis of bone mineral density (g/cc) (*n* = 6). (D) Representative images of H&E staining of rat calvarial bone defects. Scale bar = 1 mm. *P < .05, **P < .01, ***P < .001.

Compared with the controls, O-HDPSCs pretreated with OSU-T315 for 3 days showed elevated mRNA expression of osteogenic markers (Alp, Runx2, and Bmp2) and odontoblast marker genes (Dmp1 and Dspp) after induced by osteogenic differentiation medium for 9 days (Figure 6A). To evaluate bone regeneration in vivo, rat calvarial bone defects model were used. The 3-dimensional imaging by a micro-CT scan showed that O-HDPSCs pretreated with 500 nM OSU-T315 had remarkably stronger bone regeneration (Figure 6B). Quantitative analysis of bone mineral density showed that the bone density of O-HDPSCs pretreated with 500 nM OSU-T315 was higher than that of the other 2 groups (*P* < .05), which was similar to that of Y-HDPSCs (Figure 6C). The defect area of the blank group was mostly covered by fibrous tissue (Figure 6D). The group of O-HDPSCs pretreated with 500 nM OSU-T315 showed robust new bone formation and coverage of almost all the defect areas (Figure 6D), indicating that OSU-T315 could promote O-HDPSCs bone regeneration.

## Discussion

Our study showed that the proliferation and osteoblastic differentiation of HDPSCs isolated from old donors were impaired, which was consistent with other results.^14,27,28^ Moreover, the expression of senescence-related genes and SA-β-gal activity was increased in O-HDPSCs. In summary, with the appearance of cellular senescence characteristics, the proliferation and osteogenic differentiation of HDPSCs were impaired. Consistent with the features of HDPSCs isolated from donors of different age spans, our heatmap showed a higher potential of proliferation, differentiation, and migration in CCP1.

The enrichment of KEGG showed that significant proteins positively correlated with age were enriched in the response to mTOR signaling pathway. A previous report showed that mTOR signaling pathway activation caused excessive protein synthesis, resulting in impaired protein balance and accelerated aging.^15^ Autophagy, which degrades excess cellular proteins and damaged organelles from cells, declines with aging.^16,28^ Our study showed that the expression levels of kinases (ILK and MTOR) and 2 transcription factors (STAT1 and STAT6) were significantly positively correlated with age. STAT1 and STAT6 may be regulated by the phosphorylation signal of mTOR kinase, leading to aging. ILK overexpression, with subsequent mTOR activation, may be the molecular mechanism involved in hyperphosphatemia-induced senescence.^22^ Recent studies have reported that STAT1 regulated T-cell senescence.^29^ Inhibition of both the ILK and mTOR signaling pathways alleviated the senescence of HDPSC, affecting STAT1 signaling activation. Our results suggested that the ILK/AKT/mTOR/STAT1 signaling pathway may be the main factor regulating the senescence of HDPSC. However, the underlying mechanisms need to be further elucidated, and whether any of the differential genes found in proteomics play an important role in HDPSC senescence remains to be explored.

Considering the heterogeneity of individual dental pulp stem cells,^30,31^ we expanded the sample size. Moreover, the samples showed a relatively consistent mean distribution for the protein abundances, confirming high technical reproducibility. Our study has thus far established a comprehensive landscape of HDPSCs at the proteomic level, and we revealed that the senescence of HDPSCs is a multifaceted process that involves DNA replication, differentiation, migration, immunity, and autophagy. Consistent with proteomic results, we found that the proliferation, migration, and osteoblastic differentiation capacity of senescent HDPSCs were impaired with aging. Feng et al showed DPSCs showed characteristics of senescence as a function of age.^14^

HDPSCs are recognized as an excellent source of cells for regenerative therapies because of their high potential of proliferation and multilineage differentiation.^32-34^ However, a principal limitation of HDPSC-mediated therapy is that the self-renewal ability and regenerative potential were decreased in HDPSCs from elderly people.^6,35^ Moreover, no more than 4-7 population doublings of MSCs are recommended for regenerative therapies.^36^ Elucidating the molecular mechanisms of HDPSC senescence is vital for improving the therapeutic effects of HDPSC-mediated regenerative medicine. Liu et al reported that metformin alleviated HDPSC senescence by targeting CAB39 and its potential as a therapeutic target for senescence treatment.^37^ Our study elucidates that OSU-T315 alleviates HDPSC senescence and provides a basis for potential clinical application of OSU-T315 in delaying senescence. After pretreatment with inhibitors, the succeeding passage of DPSCs was used for induction in vitro or transplantation/engraftment in vivo. The regenerative properties of the succeeding passage of inhibitors pre-treated HDPSCs were enhanced. Compared with direct application of inhibitors in vivo, pretreatment with inhibitors is safer.

Previous studies reported that treatment with rapamycin in aged mice was sufficient to reverse periodontal disease.^38^ Effects of rapamycin on osteogenic differentiation is controversial. It was reported that rapamycin promoted osteogenic differentiation of BMSC and maxillary sinus membrane stem cells.^39,40^ In contrast, rapamycin inhibited osteogenesis and calcium deposition of cartilage endplate stem cells.^41^ We have identified that pretreatment with OSU-T315 promoted the osteogenesis of O-HDPSCs in vitro and in vivo, improving the potential application of O-HDPSCs in regenerative medicine. Whether treatment with OSU-T315 was sufficient to reverse periodontal disease are needed to identify.

The proliferation and osteoblastic differentiation potential of HDPSCs were impaired with aging. The senescence of HDPSC appears to be regulated by the ILK/AKT/mTOR/STAT1 signaling pathway. Treatment with OSU-T315 rejuvenated the senescence of HDPSC by targeting ILK via regulating AKT/mTOR/STAT1 signaling pathway.

## Supplementary material

Supplementary material is available at *Stem Cells* online.

sxae047_suppl_Supplementary_Materials

## Data Availability

The data used and/or analyzed during the current study are contained within the manuscript or available from the corresponding author on reasonable request.
