# Supplementary material for: Integrin-linked kinase control dental pulp stem cell senescence via the mTOR signaling pathway
Source: Stem Cells. 2024 Sep 2;42(10):861–73. doi: 10.1093/stmcls/sxae047 (PMC11464141; doi:10.1093/stmcls/sxae047)
Supplement: sxae047_suppl_Supplementary_Materials [file sxae047_suppl_supplementary_materials.pdf]

Integrin-linked kinase control dental pulp stem cell senescence via the mTOR signaling pathway

Appendix Table 1: Details of different age donors

Donor	Age (years old)	Gender
1	15	Female
2	15	Male
3	16	Male
4	17	Male
5	17	Female
6	19	Female
7	20	Female
8	22	Male
9	23	Male
10	28	Female
11	32	Male
12	33	Male
13	36	Male
14	38	Female
15	38	Female
16	40	Male
17	42	Female
18	44	Female
19	45	Male
20	46	Male
21	51	Female
22	55	Male
23	56	Female
24	65	Female
25	69	Male

Appendix Table 2: The primers used in the study

Gene	Sequence 5' to 3'
GAPDH-F	GGAGCGAGATCCCTCCAAAAT
GAPDH-R	GGCTGTTGTCATACTTCTCATGG
P16-F	TGCCCAACGCACCGAATAGT
P16-R	CAGCAGCTCCGCCACTCG
P21-F	TGTCCGTCAGAACCCATGC
P21-R	AAAGTCGAAGTTCCATCGCTC
P53-F	AATCTACTGGGACGGAACAGCTTTGAGG
P53-R	GGAGAGGAGCTGGTGTTGTTGGG
IL6-F	GCCCAGCTATGAACTCCTTCT
IL6-R	GAAGGCAGCAGGCAACAC
IL8-F	ACTGAGAGTGATTGAGAGTGGAC
IL8-R	AACCCTCTGCACCCAGTTTTC
MMP13-F	ACTGAGAGGCTCCGAGAAATG
MMP13-R	GAACCCCGCATCTTGGCTT
ALP-F	GTGAACCGCAACTGGTACTC
ALP-R	GAGCTGCGTAGCGATGTCC
RUNX2-F	CAGACCAGCAGCACTCCATAT
RUNX2-R	CAGCGTCAACACCATCATTC
BMP2-F	ACTACCAGAAACGAGTGGGAA
BMP2-R	GCATCTGTTCTCGGAAAACCT
DSPP-F	GCATTCAGGGACAAGTAAGCA
DSPP-R	CTTGGACAACAGCGACATCCT
DMP1-F	AGCCATTCTGAGGAAGACGA
DMP1-R	TGTTGTGATAGGCATCAACTGTTA
SOX2-F	GCCGAGTGGAAACTTTTGTCG
SOX2-R	GGCAGCGTGTACTTATCCTTCT
GFAP-F	CTGTTGCCAGAGATGGAGGTT
GFAP-R	TCATCGCTCAGGAGGTCCTT
GAP43-F	GGCCGCAACCAAAATTCAGG
GAP43-R	CGGCAGTAGTGGTGCCTTC

Appendix Table 3: Top 10 KEGG pathways for each CCP

	CCP		
Top 10 KEGG pathways	CCP1	CCP2	CCP3
1	DNA replication	ECM-receptor interaction	Yersinia infection
2	Mismatch repair	Hypertrophic cardiomyopathy	Ubiquitin mediated proteolysis
3	Nucleotide excision repair	Dilated cardiomyopathy	Fc gamma R-mediated phagocytosis
4	RNA polymerase	Arrhythmogenic right ventricular cardiomyopathy	Pathogenic Escherichia coli infection
5	Spliceosome	Focal adhesion	Amino sugar and nucleotide sugar metabolism
6	Steroid biosynthesis	Lysosome	Platelet activation
7	Cell cycle	Human papillomavirus infection	Regulation of actin cytoskeleton
8	Ribosome biogenesis in eukaryotes	Proteoglycans in cancer	Human cytomegalovirus infection
9	RNA degradation	Regulation of actin cytoskeleton	Vascular smooth muscle contraction
10	Terpenoid backbone biosynthesis	PI3K-Akt signaling pathway	Legionellosis

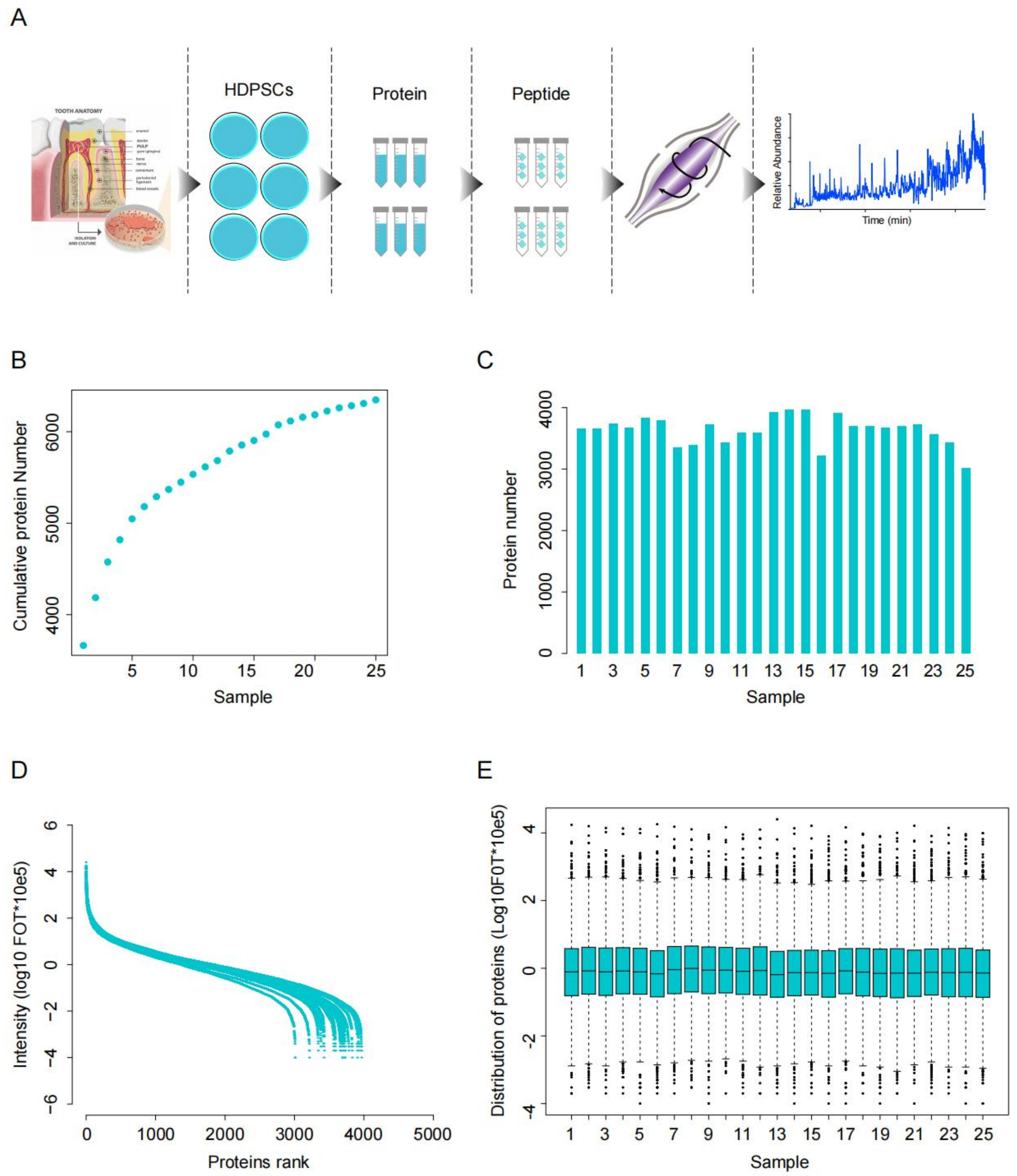

Appendix Figure 1. Proteomic landscape of HDPSCs samples. **(A)** Overview of the experimental design for proteomics analyses. **(B)** Cumulative number of proteins

identified as a function of sample numbers. (**C)** Number of proteins identified in 25 samples. **(D)** Overview of the proteomics profile of HDPSCs samples. The dynamics of protein abundances identified in 25 samples are shown. Proteins were quantified as a normalized intensity-based fraction of total (FOT) value and log10 transformed. The highest-abundance and lowest-abundance proteins are shown. **(E)** Distribution of protein abundance presented as median intensity in 25 samples.

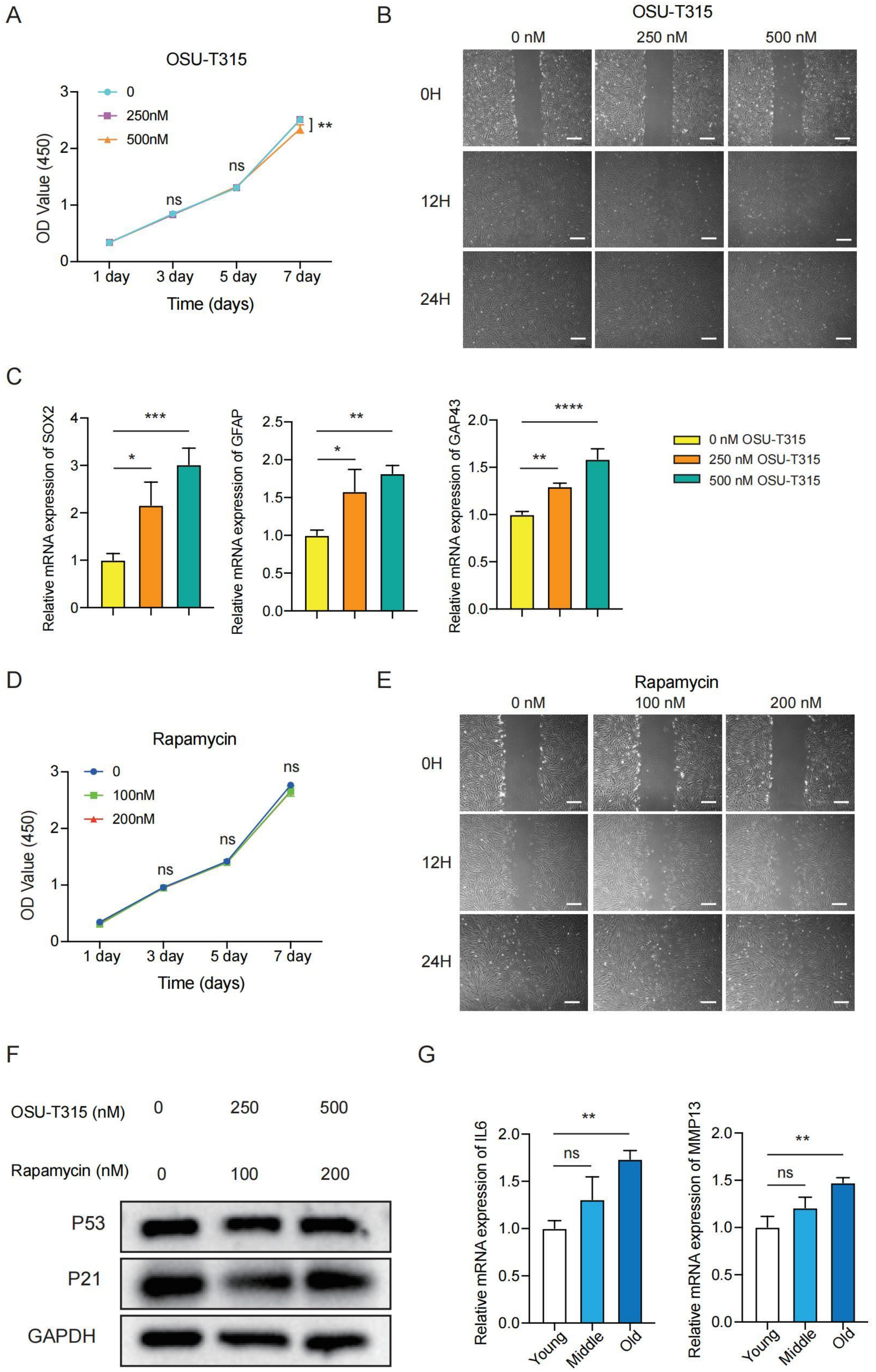
A
OSU-T315
0
250nM
500nM
OD Value (450)
Time (days)
1 day
3 day
5 day
7 day
ns
**
B
OSU-T315
0 nM
250 nM
500 nM
0H
12H
24H
C
Relative mRNA expression of SOX2
Relative mRNA expression of GFAP
Relative mRNA expression of GAP43
0 nM OSU-T315
250 nM OSU-T315
500 nM OSU-T315
D
Rapamycin
0
100nM
200nM
OD Value (450)
Time (days)
E
Rapamycin
0 nM
100 nM
200 nM
F
OSU-T315 (nM) 0 250 500
Rapamycin (nM) 0 100 200
P53
P21
GAPDH
G
Relative mRNA expression of IL6
Relative mRNA expression of MMP13
Young
Middle
Old

Appendix Figure 2. Cell function changes of O-HDPSCs pretreatment with inhibitors (OSU-T315 and Rapamycin). **(A)** Results of the CCK-8 assay of HDPSCs pretreatment with OSU-T315 (n=3). **(B)** Representative images of migration of HDPSCs pretreatment with OSU-T315. Scale bar = 100 μm. **(C)** Quantitative RT-PCR results of mRNA expression of SOX2, GFAP and GAP43 in O-HDPSCs pretreated with and without OSU-T315 induced in neurogenic induction medium for up to 7 days (n=3). **(D)** Results of the CCK-8 assay of HDPSCs pretreatment with OSU-T315 (n=3). **(E)** Representative images of migration of HDPSCs pretreatment with Rapamycin. Scale bar = 200 μm. **(F)** O-HDPSCs treated with serial concentrations of OSU-T315 and Rapamycin for 3 days were subjected to western blot analysis. **(G)** Quantitative RT-PCR results of mRNA expression of IL6 and MMP13 in different age groups (n=3). *$p<0.05$. **$p<0.01$, ***$p<0.005$.
